# Cervical Cancer Screening Prevalence and Predictors Among Women Aged 25–49 Years in Ghana: A Cross‐Sectional Study

**DOI:** 10.1002/hsr2.71971

**Published:** 2026-03-02

**Authors:** Frank Kyei‐Arthur, Martin Wiredu Agyekum, Grace Frempong Afrifa‐Anane, Nurudeen Alhassan, Nuworza Kugbey, Kofi Mensah Nyarko

**Affiliations:** ^1^ Department of Environment and Public Health University of Environment and Sustainable Development Somanya Eastern Region Ghana; ^2^ Legon Centre for Education Research and Policy University of Ghana Legon Greater Accra Ghana; ^3^ African Institute for Development Policy Lilongwe Central Region Malawi

**Keywords:** cervical cancer, cervical cancer screening, Ghana, predictors, prevalence, sub‐Saharan Africa

## Abstract

**Background and Aims:**

Cervical cancer can be prevented by early detection through regular screening. The American Cancer Society advocates the initiation of cervical cancer screening from 25 years of age. Few studies in Ghana have used nationally representative data to examine the prevalence and predictors of cervical cancer screening (CCS). Therefore, this study examined the prevalence and predictors of CCS among women aged 25–49 years in Ghana.

**Methods:**

This study used cross‐sectional secondary data from the 2022 Ghana Demographic and Health Survey (GDHS), which involved 9510 women. Binary logistic regression was used to examine the predictors of CCS. All variables were considered statistically significant at a 95% confidence interval (*p*‐value < 0.05).

**Results:**

Overall, the prevalence of CCS was 6.90% (95% CI = 6.39%–7.41%). Women's age, educational level, marital status, parity, ecological zone, age at menarche, HIV testing, frequency of listening to the radio, and interaction between age and parity were predictors of CCS. On the one hand, women who had 6 or more children (95% CI = 0.04–0.54), those aged 45–49 years with 4–5 children (95% CI = 0.04–0.98), and those with menarche at ≥ 15 years (95% CI = 0.59–0.97), were less likely to screen for cervical cancer. On the other hand, women with tertiary education (95% CI = 1.07–10.84), those from the Northern ecological zone (95% CI = 1.34–3.23), and those who had ever tested for HIV (95% CI = 1.49–3.36), were more likely to screen for cervical cancer.

**Conclusion:**

The prevalence of CCS is low in Ghana, and there is an urgent need for policymakers to enhance health promotion campaigns on cervical cancer screening to increase the uptake of screening services.

## 1 Introduction

Cervical cancer is a significant global public health issue affecting the health and well‐being of women. It is the fourth most common cancer in women and the seventh most common cancer [1, 2]. Globally, about 604,127 women were diagnosed with cervical cancer, and 341,831 died from the condition in 2020 [1]. Cervical cancer disproportionately affects women from poor households, with at least 80% of deaths occurring in developing countries [3]. However, the disease is preventable by early detection through regular screening. Screening seeks to identify precancerous cellular changes on the cervix that may become cervical cancer if they are not appropriately treated [2]. Evidence shows that most patients in developing countries present cervical cancer at health facilities in the late stages, making it challenging to treat [4, 5]. Studies have documented that late presentation of cervical cancer at health facilities could be a result of several factors, such as delay in screening, poor knowledge about the disease, lack of awareness of the screening process, and weak healthcare systems, where not all health facilities have screening equipment [3, 6]. The American Cancer Society recommends initiating cervical cancer screening from the age of 25 years [7].

In the year 2022, cervical cancer emerged as the second most common cancer among women in Ghana [8]. In addition, Ghana reported a total of 3072 newly diagnosed cases of cervical cancer and a mortality of 1815 cases in 2022 [8]. According to Bruni et al. [1], in Ghana, it is estimated that 27.4 per 100,000 women were diagnosed with cervical cancer in 2020, while 17.8 per 100,000 women died from cervical cancer. Also, an estimated 5‐year prevalence of cervical cancer cases (2018–2022) in Ghana was 7,889 (49.4 per 100,000 women) [1].

In 2001, the screening and testing of cervical cancer in Ghana were introduced and fully incorporated into the National Reproductive Health Service Delivery Guidelines [9, 10]. Yet, evidence shows that few women undergo screening to know their status [3, 11]. For instance, Ayanore et al.'s [11] study reported that 12.0% of women aged 18 years or older reported being screened for cervical cancer. In comparison, Calys‐Tagoe et al.'s [3] study revealed that 8.3% of women aged 18 years or older had undergone cervical cancer screening.

Though there are studies on cervical cancer screening in sub‐Saharan African countries [12, 13, 14, 15], evidence from these studies has shown that some predictors of cervical cancer screening are context‐specific [14]. Bekalu et al.'s [14] study in Ethiopia found that the context‐specific predictors of cervical cancer were a bad obstetrics history, a history of gynecologic problems, awareness of cervical cancer, and a family history of cervical cancer. Also, Okyere et al.'s [13] study in Côte d'Ivoire found that the context‐specific predictors of cervical cancer were age at menarche, history of sexually transmitted infections, and health insurance. These evidences necessitates the need to conduct country‐specific studies to determine context‐specific predictors of cervical cancer screening so that context‐specific interventions can be developed to address the poor utilisation of cervical cancer screening services.

In Ghana, there are limited studies on cervical cancer screening [3, 11, 16, 17, 18]. These studies have primarily focused on barriers to cervical cancer screening [16, 17] and predictors of cervical cancer screening [3, 11, 17, 19]. Predictors of cervical cancer screening included age, women's education, father's education, marital status, mother's employment, wealth status, reading newspapers, and satisfaction and involvement with healthcare [3, 11, 17, 19]. For instance, Calys‐Tagoe et al.'s [3] study using the World Health Organization's (WHO) multi‐country Study on AGEing and adult health (SAGE) wave 2 found that satisfaction and involvement with healthcare and marital status were significant predictors of cervical cancer screening in Ghana.

There are limited studies in Ghana that examined predictors of cervical cancer screening, with few using nationally representative samples [3]. To bridge this gap, this study examined the prevalence and predictors of cervical cancer screening among women aged 25–49 years in Ghana using the 2022 Ghana Demographic and Health Survey (GDHS).

## 2 Methods

### 2.1 Study Design and Description

This study used nationally representative cross‐sectional data from the 2022 GDHS conducted by the Ghana Statistical Service [20]. The GDHS used a two‐stage stratified sampling design to select households. The first stage involved selecting 618 primary sampling units or clusters, stratified by the 16 administrative regions and rural‐urban status. A household listing was conducted in all chosen clusters to develop a list of households' frame for each cluster, from which 30 households were selected to participate in the study. The study was restricted to women aged 25 years and above.

Ethical clearance was not sought for this study since it was an analysis of secondary data (2022 GDHS). The ICF Institutional Review Board and the Ghana Health Service approved the 2022 GDHS. A detailed description of the ethical issues regarding the DHS can be assessed at https://dhsprogram.com/methodology/Protecting-the-Privacy-of-DHS-Survey-Respondents.cfm. Informed consent was received from all respondents, and participation in the study was voluntary. Additionally, the data collection for the 2022 GDHS adhered to the principles outlined in the Declaration of Helsinki.

### 2.2 Study Setting

Ghana is a lower‐middle‐income country located in West Africa. It is bounded to the north by Burkina Faso, the south by the Gulf of Guinea, the east by Togo and the west by Côte d'Ivoire. Ghana has a population of 33.7 million in 2025, and it is estimated to have a population of 44.5 million by 2040 and 52.5 million by 2050 [21]. Ghana has 16 administrative regions, and Accra, located in the Greater Accra region, is the capital of Ghana.

### 2.3 Measurement of Variables

#### 2.3.1 Dependent Variable

The outcome variable for the study was the utilisation of cervical cancer screening. Cervical cancer screening utilisation was measured by asking women whether a healthcare provider had ever tested them for cervical cancer. Information on the type of cervical cancer screening test received by women was not collected in the 2022 GDHS [20]. Cervical cancer screening utilisation was categorised as “Yes” and “No.” Those who had ever been screened for cervical cancer by a healthcare provider were categorised as “Yes.” In contrast, those who had never been screened for cervical cancer by a healthcare provider were categorised as “No.” The same question has been used in previous studies to measure cervical cancer screening utilisation [12, 13].

### 2.4 Predictor Variables

The predictor variables included in the analyses were identified based on a review of the literature [3, 11, 12, 13, 17]. The predictor variables included, age of the women, which was obtained in completed years and classified into broad age groupings (25–29 years, 30–34 years, 35–39 years, and 40–44 years and 45–49 years) based on the reproductive structure of the population, educational level (no education, primary, Junior Secondary School (JSS)/Middle, secondary, and tertiary), marital status (never married, married, cohabiting and formerly in a union), parity (no child, 1 child, 2–3 children, 4–5 children, and 6 and more children), and religion (Christian, Muslims, and other religious groups). Other predictor variables were place of residence (urban and rural), National Health Insurance Sheme (NHIS) (valid and not valid), age at first sex (below 18 years, and 18 years and above), age at menarche (below 15 years, and 15 years and above), HIV tested (yes and no), had STI in the last 12 months (no and yes), frequency of reading newspaper (not at all, less than once a week and at least once a week), and frequency of listening to the radio (not at all, less than once a week and at least once a week). Socio‐economic status reflects a household's living standard, which can be indicated by ownership of household items [22] and is reclassified into poor, middle, and rich categories. Women from households with middle status have better living standards compared to women from households with poor status. Regarding ecological zone, Ghana has 16 administrative regions, which has been reclassified into three ecological zones: coastal (Eastern, Ashanti, Ahafo, Bono, and Bono East), middle (Oti, Volta, Greater Accra, Central, Western and Western North regions) and northern (Upper East, Upper West, Northern, North East and Savannah regions) zones. Furthermore, interaction variables (parity and socio‐economic status, education and socio‐economic status, and age and parity) were created to understand how the interactions between these variables influence cervical cancer screening.

### 2.5 Data Analysis

The data was analysed using descriptive and inferential statistics. The variables were presented using frequencies and percentages. Multivariate analysis, specifically binary logistic regression, was employed to examine the predictors of cervical cancer screening in Ghana, as the dependent variable was dichotomous. The odds ratio was used to measure the relationship between the dependent and predictor variables. The review of the literature on cervical cancer screening helped us to ascertain which variables should be included in the binary logistic regression model.

The data was analysed using Stata/MP version 17 software. The data were weighted, and the primary sampling unit and sample strata for sampling errors were used to create the svy command, which accounts for clusters or stratification to make the estimate representative and provide a more accurate statistical estimate. All variables were considered statistically significant at a 95% confidence interval (*p*‐value < 0.05).

In this study, we performed the Hosmer–Lemeshow goodness‐of‐fit test to assess the fitness of our binary logistic regression model. The Hosmer–Lemeshow goodness‐of‐fit test showed a *p*‐value of 0.160, which indicates that the model fits well, as there is no statistical difference between the observed and expected values. The study also examined multicollinearity using variance inflation factors (VIFs) to assess the strength of the correlations among the predictor variables. The VIF values for all predictor variables were less than 5, indicating that they are moderately correlated (See Supporting Information Tables S1, S2 and S3 in supporting material). Since the predictor variables were theoretically relevant to the study, they were included in the binary logistic regression analysis. Furthermore, a sensitivity analysis was conducted to determine the unweighted and weighted prevalence estimates of cervical cancer screening. Missing cases for cervical cancer and other independent variables were excluded using the listwise deletion.

## 3 Results

### 3.1 Background Characteristics of Respondents

Table 1 presents the socio‐demographic characteristics of respondents. Out of the 9,510 women included in this study, a higher proportion (48.50%) of the women were within the younger age population. Specifically, about 25.00% were 25–29 years, and 23.40% were 30–34 years. One‐third (30.40%) of the women had no formal education, 29.70% had JSS/Middle education, and the lowest proportion, 11.10%, had tertiary education. Regarding marital status, slightly less than two‐thirds (63.20%) of the women were married, a higher proportion (33.60%) had 2–3 children, and the smallest proportion (8.90%) had no children. The majority (68.90%) of the women were affiliated with the Christian religion, and 45.30% belonged to the poor socio‐economic status. In addition, 42.60% of women lived in the middle ecological zone, while half (50.50%) of the women lived in rural areas.

**Table 1:** Socio‐demographic characteristics of respondents.

Socio‐demographic characteristics	Frequency	Percentage
**Age of woman**		
25–29 years	2386	25.10
30–34 years	2228	23.40
35–39 years	2021	21.30
40–44 years	1646	17.30
45–49 years	1229	12.90
**Educational level**		
No education	2893	30.40
Primary	1438	15.10
JSS/Middle	2821	29.70
Secondary	1302	13.70
Tertiary	1056	11.10
**Marital status**		
Never married	1003	10.60
Married	6014	63.20
Living with a partner	1333	14.00
Formerly married	1160	12.20
**Parity**		
No child	844	8.90
1 child	1223	12.90
2–3 children	3194	33.60
4–5 children	2623	27.60
6 or more children	1626	17.10
**Religion**		
Christian	6556	68.90
Muslims	2515	26.50
Other	439	4.60
**Socio‐economic status**		
Poor	4309	45.30
Middle	1834	19.30
Rich	3367	35.40
**Ecological zone**		
Coastal	2286	24.00
Middle	4050	42.60
Northern	3174	33.40
**Place of residence**		
Urban	4705	49.50
Rural	4805	50.50

Table 2 shows the health‐related/behavioural characteristics of respondents. Seven (70.00%) out of 10 women had valid NHIS cards, while half (50.30%) had their first sex before 18 years. Slightly less than two‐thirds (63.30%) of the women had their menarche 15 years and above, 67.80% of the women had tested for HIV, and about 6.00% had sexually transmitted infections in the last 12 months. Moreover, 92.70% of the women had not read the newspaper at all, while approximately 40.00% and 54.30% of the women listened to the radio and watched television at least once a week, respectively.

**Table 2:** Health‐related/behavioural characteristics of respondents.

Health‐related/behavioural characteristics	Frequency	Percentage
**NHIS**		
Valid	6653	70.00
Not valid	2857	30.00
**Age at first sex**		
Below 18 years	4780	50.30
18 years and above	4730	49.70
**Age at menarche**		
Below 15 years	3486	36.70
15 years and above	6024	63.30
**HIV tested**		
No	3064	32.20
Yes	6446	67.80
**Had an STI in the last 12 months**		
No	8932	93.90
Yes	578	6.10
**Frequency of reading the newspaper**		
Not at all	8811	92.70
Less than once a week	463	4.90
At least once a week	236	2.50
**Frequency of listening to the radio**		
Not at all	3452	36.30
Less than once a week	2,183	23.00
At least once a week	3,875	40.80
**Frequency of watching television**		
Not at all	2931	30.80
Less than once a week	1419	14.90
At least once a week	5160	54.30

### 3.2 Prevalence of Cervical Cancer Screening

**Figure 1:**
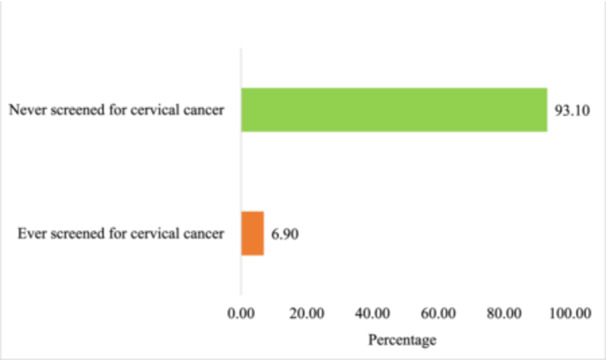
Prevalence of cervical cancer screening among women aged 25–49 years in Ghana, based on data from the 2022 GDHS. The bar graph illustrates whether respondents have ever screened for cervical cancer or not.

Figure 1 shows the prevalence of cervical cancer screening. For Figure 1, the prevalence of cervical cancer screening was 6.90% (95% CI = 6.39%–7.41%). This means 6.90% of women aged 25–49 years in Ghana have ever been screened for cervical cancer. The sensitivity tests assessing the unweighted and weighted prevalence estimates of cervical cancer screening revealed that the unweighted prevalence was lower at 6.40%, while the weighted estimate was slightly higher at 6.90%. This difference reflects the impact of sampling weights and design effects. Survey weights enhance the representativeness of the prevalence estimates for the general population and adjust for the unequal probability of selection.

### 3.3 Predictors of Cervical Cancer Screening

Table 3 illustrates the results of the binary logistic regression models examining factors associated with cervical cancer screening in Ghana. The results show that the age of women, educational level, marital status, parity, ecological zone, age at menarche, HIV testing, frequency of listening to the radio and the interaction between age and parity were significant predictors of cervical cancer screening.

**Table 3:** Estimated odds ratios of predictors of cervical cancer screening.

Variable	aOR	95% CI		*p*‐value
**Age of woman**				
*25–29 years (Ref)*	1.00			
30–34 years	1.96	1.03	3.74	0.042
35–39 years	1.11	0.33	3.73	0.871
40–44 years	4.32	1.45	12.89	0.009
45–49 years	9.52	3.20	13.36	< 0.001
**Educational level**				
*No education (Ref)*	1.00			
Primary	1.28	0.65	2.51	0.480
JSS/Middle	1.58	0.83	3.01	0.167
Secondary	1.57	0.50	5.01	0.441
Tertiary	3.40	1.07	10.84	0.038
**Marital status**				
*Never married (Ref)*	1.00			
Married	1.68	1.10	2.55	0.015
Living with a partner	1.69	1.04	2.76	0.035
Formerly married	1.88	1.13	3.15	0.016
**Parity**				
*No child (Ref)*	1.00			
1 child	0.35	0.09	1.33	0.124
2–3 children	0.71	0.20	2.52	0.596
4–5 children	0.38	0.08	1.78	0.218
6 and more children	0.14	0.04	0.54	0.004
**Religion**				
*Christian (Ref)*				
Muslims	1.06	0.74	1.50	0.761
Other	0.51	0.18	1.43	0.199
**Socio‐economic status**				
*Poor (Ref)*	1.00			
Middle	0.81	0.16	4.15	0.796
Rich	1.88	0.43	8.14	0.397
**Ecological zone**				
*Coastal (Ref)*	1.00			
Middle	1.35	0.97	1.87	0.072
Northern	2.08	1.34	3.23	0.001
**Place of residence**				
*Urban (Ref)*	1.00			
Rural	0.79	0.57	1.10	0.168
**NHIS**				
*Valid (Ref)*	1.00			
Not valid	0.76	0.53	1.11	0.160
**Age at first sex**				
*Below 18 years (Ref)*	1.00			
18 years and above	1.06	0.83	1.36	0.645
**Age at menarche**				
*Below 15 years (Ref)*	1.00			
15 years and above	0.76	0.59	0.97	0.026
**HIV tested**				
*No (Ref)*	1.00			
Yes	2.24	1.49	3.36	< 0.001
**Had an STI in the last 12 months**				
*No (Ref)*	1.00			
Yes	1.06	0.68	1.63	0.805
**Frequency of reading the newspaper**				
*Not at all (Ref)*	1.00			
Less than once a week	1.43	0.99	2.05	0.055
At least once a week	1.23	0.74	2.04	0.425
**Frequency of listening to the radio**				
*Not at all (Ref)*	1.00			
Less than once a week	1.11	0.81	1.51	0.528
At least once a week	1.35	1.02	1.80	0.038
**Frequency of watching television**				
*Not at all (Ref)*	1.00			
Less than once a week	1.19	0.79	1.78	0.408
At least once a week	1.20	0.77	1.86	0.414
**Parity x socio‐economic status**				
*No child x Middle (Ref)*	1.00			
1 child x Middle	3.77	0.75	19.11	0.108
1 child x Rich	3.12	0.85	11.41	0.085
2–3 children x Middle	1.36	0.34	5.54	0.665
2–3 children x Rich	1.38	0.43	4.44	0.588
4–5 children x Middle	1.67	0.39	7.17	0.488
4–5 children x Rich	2.04	0.64	6.53	0.228
6 and more children x Middle	2.01	0.42	9.59	0.378
6 and more children x Rich	1.55	0.38	6.31	0.543
**Education x socio‐economic status**				
*No education x Middle (Ref)*	1.00			
Primary x Middle	1.63	0.57	4.60	0.359
Primary x Rich	0.36	0.11	1.18	0.090
JSS/Middle x Middle	1.00	0.36	2.79	0.996
JSS/Middle x Rich	0.40	0.16	1.01	0.053
Secondary x Middle	0.89	0.22	3.67	0.873
Secondary x Rich	0.73	0.18	2.96	0.657
Tertiary x Middle	0.82	0.13	5.21	0.836
Tertiary x Rich	0.70	0.21	2.34	0.557
**Age x Parity**				
*25–29 years x 1 child (Ref)*	1.00			
30–34 years x 1 child	0.69	0.29	1.61	0.389
30–34 years x 2–3 children	0.52	0.23	1.18	0.117
30–34 years x 4–5 children	0.62	0.17	2.24	0.467
30–34 years x 6 and more children	2.60	0.64	10.55	0.182
35–39 years x 1 child	1.00	0.23	4.41	0.999
35–39 years x 2–3 children	1.08	0.27	4.32	0.912
35–39 years x 4–5 children	1.83	0.35	9.68	0.476
35–39 years x 6 and more children	7.99	1.50	42.64	0.015
40–44 years x 1 child	0.28	0.07	1.17	0.081
40–44 years x 2–3 children	0.42	0.12	1.44	0.169
40–44 years x 4–5 children	0.56	0.11	2.75	0.474
40–44 years x 6 and more children	1.11	0.23	5.32	0.899
45–49 years x 1 child	0.52	0.12	2.24	0.377
45–49 years x 2–3 children	0.25	0.07	0.88	0.031
45–49 years x 4–5 children	0.20	0.04	0.98	0.047

Abbreviations: aOR, adjusted odds ratio; CI, confidence interval; Ref, reference category.

Women who were aged 30–34 years [aOR = 1.96; 95% CI = 1.03–3.74], 40–44 years [aOR = 4.32; 95% CI = 1.45–12.89], and 45–49 years [aOR = 9.52; 95% CI = 3.20–13.36] were more likely to go for cervical cancer screening than those who were 25–29 years. Women who had a tertiary education [aOR = 3.40; 95% CI = 1.07–10.84] were more likely to go for cervical screening than those with no formal education. Women who were married, living with a partner, and formerly married were 1.68 [CI = 1.10–2.55], 1.69 [CI = 1.04–2.76], and 1.88 [CI = 1.13–3.15] times, respectively, more likely to screen for cervical cancer compared to those who were never married. Women who had 6 or more children [aOR = 0.14; 95% CI = 0.04–0.054] were less likely to go for cervical cancer screening than those with one child.

In addition, women living in the Northern ecological zone [aOR = 2.08; 95% CI = 1.34–3.23] were more likely to go for cervical cancer screening than those living in the Coastal zone. Women who had their menarche 15 years and above [aOR = 0.76; 95% CI = 0.59–0.97] were less likely to go for cervical cancer screening than those who had their menarche below 15 years. Furthermore, women who had tested for HIV [aOR = 2.24; 95% CI = 1.49–3.36] were more likely to go for cervical cancer than those who had never tested for HIV. Women who listened to the radio at least once a week [aOR = 1.35; 95% CI = 1.02–1.80] were more likely to go for cervical cancer than those who did not listen to the radio.

Moreover, women aged 35–39 years with 6 or more children [aOR = 7.99; 95% CI = 1.50–42.64] were more likely to go for cervical cancer screening than those aged 25–29 years with a child. However, women aged 45–49 years with 2–3 children [aOR = 0.25; 95% CI = 0.07–0.88] were less likely to go for cervical cancer screening than those aged 25–29 years with a child. Similarly, women aged 45–49 years with 4–5 children [aOR = 0.20; 95% CI = 0.04–0.98] were less likely to go for cervical cancer screening than those aged 25–29 years with a child.

## 4 Discussion

This study examined the prevalence and predictors of cervical cancer screening among women aged 25–49 years in Ghana using data from the 2022 GDHS. The prevalence of cervical cancer screening was 6.4% (95% CI = 6.0%–6.9%), which is lower than the prevalence reported in Côte d'Ivoire (7.5%) [13], Ghana (7.3%, 8.3%, and 12.0%) [3, 11, 19], Ethiopia (19.4%) [14], and Cameroon (34.0%) [15]. However, the prevalence of cervical cancer screening reported in this study was higher than the prevalence reported in Ghana (3.0%) [17] and Cameroon (3.5%) [12]. The difference in the prevalence of cervical cancer screening rates can be attributed to several factors, including the sample population, sample size, nationally representative data, the years of data collection, and the measurement of the dependent variable. Specifically, the prevalence of cervical cancer screening reported in our study differs from previous studies in Ghana due to differences in the age category of respondents and the use of nationally representative data. For instance, Calys‐Tagoe et al. [3] and Ayanore et al. [11] studies in Ghana used the WHO Study on Global AGEing and Adult Health (SAGE) dataset (a nationally representative data), which focused on women aged 18 years or older, while our study using the 2022 GDHS (a nationally representative data) focused on women aged 25 to 49 years. Also, Ampofo et al.'s [17] study in Ghana focused on women aged 15 to 50 years in the Ashanti Region of Ghana, while Adzigbli et al.'s [19] study in Ghana using the 2022 GDHS focused on women aged 30‐49 years. Ampofo et al. [17] and Adzigbli et al. [19] studies differed from our study in terms of the age of the respondents. Additionally, Ampofo et al.'s [17] study differed from ours in terms of the national representativeness of its data.

Regarding predictors of cervical cancer screening, women's age, educational level, marital status, parity, ecological zone, age at menarche, HIV testing, frequency of listening to the radio and the interaction between age and parity were significant predictors of cervical cancer screening. This study found that increasing age (30 years or older) was associated with screening for cervical cancer, which supports previous studies [11, 12, 13]. A plausible explanation is that increasing age elevates women's risk of developing cervical cancer [22]. Consequently, as women age, they will want to screen for cervical cancer. A study by Kahesa et al. [23] in Tanzania found that women aged 35‐59 years were more willing to accept cervical cancer screening than those aged 25‐34 years.

Women's education played a significant role in influencing women to screen for cervical cancer. Corroborating previous studies [12, 13, 19, 24], this study revealed that women with tertiary education were more likely to screen for cervical cancer than those with no formal education. Women with tertiary education are more likely to be aware of cervical cancer and its harmful consequences. Therefore, it may encourage them to take precautionary measures, including screening, to reduce their risk of getting the condition [13].

Another finding was that women who are married, living with a partner, or formerly married increased their odds of screening for cervical cancer compared to those who are never married, confirming previous studies in Ghana [3], Tanzania [23], and Cameroon [12]. Partners of women who are married or living with partners can influence their healthcare seeking behaviour [25], including the uptake of cervical cancer screening. In Ghana, married women culturally receive more social support and assistance in accessing healthcare services than unmarried women [26], which may explain why they have a higher likelihood of screening for cervical cancer than unmarried mothers.

However, this study's finding contradicts a previous study in Ghana, which found that married women were more likely to screen for cervical cancer [17]. The mixed findings regarding marital status and cervical cancer screening can be attributed to various factors, including the representativeness of data and the measurement of marital status. For instance, Ampofo et al.'s study in Ghana was conducted in the Ashanti region of Ghana, and marital status was categorised as unmarried and married. However, our data is representative of women aged 25 to 49 years in Ghana, and marital status was categorised as never married, married, living with a partner, and formerly married.

Parity was also significantly associated with cervical cancer screening. Women with higher parity (6 or more children) were less likely to screen for cervical cancer. Traditionally, mothers have higher caregiving responsibilities caring for their children compared to non‐mothers, which may hinder their utilisation of healthcare services [27], thereby reducing their odds of screening for cervical cancer. Our finding does not support a study in Ethiopia by Bekalu et al. [14], which found that multipara women were more likely to be screened for cervical cancer. Also, a study in Zimbabwe by Zibako et al. [24] found that parity was not significantly associated with cervical cancer screening. Factors such as differences in the measurement of parity and study population, could explain the difference in these findings. For example, Zibako et al.'s [24] study was facility‐based, and parity was measured as a continuous variable. In contrast, our study categorised the parity of women, and it was a community‐based study.

Our study revealed that cervical cancer screening significantly varies among different age and parity groups of women. Women aged 35–39 years with 6 or more children were more likely to screen for cervical cancer. However, women aged 45–49 years with 2–5 children were less likely to screen for cervical cancer.

Regarding ecological zones, women living in the Northern ecological zone had higher odds of screening for cervical cancer than those living in the Coastal zone. Several health interventions have been implemented in the Northern ecological zone to enhance women's healthcare seeking behaviour, notably the Community‐based Health Planning and Services (CHPS) programme [28]. The enhancement of women's healthcare‐seeking behaviour may lead to a heightened utilisation of healthcare services, including cervical cancer screening.

Contrary to the findings of Okyere et al. [13], this study found that women who experienced early menarche (before 15 years) were more likely to screen for cervical cancer. A plausible explanation is that early menarche increases a woman's risk of cervical cancer [18, 29]. Furthermore, this study found that women who had ever tested for HIV had a higher likelihood of screening for cervical cancer. In Ghana, HIV testing services are primarily conducted within healthcare facilities [30]. Therefore, women who utilise these services may encounter other healthcare services, such as cervical cancer screening. This exposure may enhance their use of these services.

Supporting a previous study [13], this study found that listening to the radio increased the odds of cervical cancer screening. Listening to the radio may increase women's awareness of cervical cancer and its preventive measures, such as screening. Consequently, it can increase their uptake of cervical cancer screening. A study by Bekalu et al. [14] found that hearing about cervical cancer increases the uptake of cervical cancer screening.

Though not statistically significant, women residing in rural areas were less likely to screen for cervical cancer than those living in urban areas. In Ghana, there are more health facilities in urban areas compared to rural areas [31], which provides more opportunity for women in urban areas to screen for cervical cancer compared to their counterparts in rural areas. The insignificant relationship between place of residence and cervical cancer screening may be attributable to the nearly equal distribution of women residing in urban areas (49.50%) and rural areas (50.50%).

Moreover, the study found that religion, socio‐economic status, NHIS, age at first sex, having an STI in the last 12 months, frequency of reading newspapers, frequency of watching television, interaction between parity and socio‐economic status, and education and socio‐economic status were not statistically significant predictors of cervical cancer screening. Further studies are needed to validate the relationship between these variables and cervical cancer screening, since they are expected to have significant relationships with cervical cancer screening. However, our study found that women from rich households were more likely to screen for cervical cancer compared to those from poor households. Women from rich households have the financial resources to access cervical cancer screening. Studies have identified financial constraints as a barrier to cervical cancer screening [16, 18, 32, 33].

### 4.1 Implications for Practice

The low rate of cervical cancer screening among women aged 25 to 49 years, an essential preventive measure, undermines Ghana's achievement of the Sustainable Development Goal Target 3.4, which seeks to reduce premature mortality from non‐communicable diseases (NCDs), including cervical cancer, by one‐third through prevention and treatment. The Ghana National Policy on Non‐Communicable Diseases highlights the promotion of routine screening as a key strategy for preventing NCDs [34]. Hence, policymakers and practitioners need to strengthen the structures aimed at promoting routine cancer screening across the country to increase the uptake of cervical cancer screening. Increased uptake of cervical cancer screening can help reduce government expenditure on cervical cancer treatment covered by the National Health Insurance Scheme [35].

Also, women with higher parity were less likely to screen for cervical cancer. Policymakers and health practitioners need to target these vulnerable women during the promotion of cervical cancer screening since they have an elevated risk of cervical cancer [36, 37]. Furthermore, our study highlighted that cervical cancer screening behaviour of women is not uniform across their different age and parity groups. These findings imply that there is a need for health professionals and policymakers to target both the age and parity of women in the design of interventions to increase the uptake of cervical cancer screening. Consequently, health promoters should use different health campaign messages to target these women, given their unique cervical cancer screening behaviours.

Moreover, this study identified women without formal education and those who have never married as vulnerable sub‐populations, as they were less likely to have cervical cancer screening. Health professionals and policymakers should specifically focus on these sub‐populations of women when designing and implementing programmes to enhance cervical cancer screening uptake.

### 4.2 Strengths and Limitations of the Study

The main strength of this study was the use of 2022 GDHS data, a nationally representative sample, to examine the prevalence and predictors of cervical cancer screening among women aged 25‐49 years in Ghana. Nevertheless, this study had some limitations. First, the study was cross‐sectional. Therefore, causality cannot be established between the dependent and predictor variables. Second, the respondents' information was self‐reported and subject to recall bias. Hence, the study's findings should be interpreted with caution. Third, the use of secondary data did not enable the researchers to examine the types of screening tests performed and the barriers to cervical cancer screening, as these were not captured in the 2022 GDHS. Future studies should examine the types of cervical cancer screening tests use by women and the barriers to cervical cancer screening to enhance the development of appropriate interventions to increase patronage of cervical cancer screening and improve the early detection of cervical cancer.

## 5 Conclusion

This study demonstrated that the prevalence of cervical cancer screening was low (6.4%) in Ghana. Consequently, there is an urgent need for policymakers to enhance health promotion campaigns on cervical cancer screening to increase its uptake. Also, these health promotion campaigns should consider the socio‐demographic (age of women, educational level, marital status, and parity), geographical (ecological zone), health‐related (age at menarche and HIV testing), and behavioural (listening to the radio) factors identified as significant predictors of cervical cancer screening in this study to enhance their effectiveness.

## Author Contributions

**Frank Kyei‐Arthur:** conceptualisation, formal analysis, visualisation, writing – original draft, writing – review and editing, validation, supervision. **Martin Wiredu Agyekum:** conceptualisation, formal analysis, visualisation, writing – original draft, writing – review and editing, validation, supervision. **Grace Frempong Afrifa‐Anane:** conceptualisation, formal analysis, visualisation, writing – original draft, writing – review and editing, validation, supervision. **Nurudeen Alhassan:** conceptualisation, visualisation, writing – original draft, writing – review and editing, validation, supervision. **Nuworza Kugbey:** conceptualisation, visualisation, writing – original draft, writing – review and editing, validation, supervision. **Kofi Mensah Nyarko:** conceptualisation, visualisation, writing – original draft, writing – review and editing, validation, supervision.

## Funding

The authors received no specific funding for this work.

## Ethics Statement

The 2022 GDHS was approved by the ICF Institutional Review Board, and the Ghana Health Service approved the secondary data. Informed consent was obtained from all respondents. Additionally, the collection of the 2022 GDHS data adhered to the principles outlined in the Declaration of Helsinki.

## Conflicts of Interest

The authors declare no conflicts of interest.

## Transparency Statement

The lead author Frank Kyei‐Arthur affirms that this article is an honest, accurate, and transparent account of the study being reported; that no important aspects of the study have been omitted; and that any discrepancies from the study as planned (and, if relevant, registered) have been explained.

## Supporting information


**Table S1:** Multicollinearity test of education, socio‐economic status and cervical cancer screening. **Table S2:** Multicollinearity test of age, parity and cervical cancer screening. **Table S3:** Multicollinearity test of parity, Socio‐economic status and cervical cancer screening.

## Data Availability

The data used for this study is freely available at https://dhsprogram.com/data/available-datasets.cfm.
