# Supplementary material for: Cervical Cancer Screening Prevalence and Predictors Among Women Aged 25–49 Years in Ghana: A Cross‐Sectional Study
Source: Health Sci Rep. 2026 Mar 2;9(3):e71971. doi: 10.1002/hsr2.71971 (PMC12953717; doi:10.1002/hsr2.71971)
Supplement: Supplementary file 1 — Table S1: Multicollinearity test of education, socio‐economic status and cervical cancer screening. Table S2: Multicollinearity test of age, parity and cervical cancer screening. Table S3: Multicollinearity test of parity, Socio‐economic status and cervical cancer screening. [file HSR2-9-e71971-s001.docx]

Table S1. Multicollinearity test of education, socio-economic status and cervical cancer screening

| **Variable** | **Coefficient** | **95% CI** | | **P-value** | **VIF** |
| --- | --- | --- | --- | --- | --- |
| **Education** |  |  |  |  |  |
| *No education (Ref)* | 1.00 |  |  |  |  |
| Primary | 0.01 | -0.01 | 0.02 | 0.427 | 1.31 |
| JSS/Middle | 0.01 | -0.01 | 0.02 | 0.316 | 1.61 |
| Secondary | 0.02 | 0.00 | 0.04 | 0.012 | 1.50 |
| Tertiary | 0.14 | 0.12 | 0.16 | < 0.001 | 1.63 |
| **Socio-economic status** |  |  |  |  |  |
| *Poor (Ref)* | 1.00 |  |  |  |  |
| Middle | 0.01 | 0.00 | 0.03 | 0.037 | 1.25 |
| Rich | 0.04 | 0.03 | 0.05 | < 0.001 | 1.67 |

CI: Confidence Interval: Ref: Reference Category

Table S2. Multicollinearity test of age, parity and cervical cancer screening

| **Variable** | **Coefficient** | **95% CI** | | **P-value** | **VIF** |
| --- | --- | --- | --- | --- | --- |
| **Age** |  |  |  |  |  |
| *25-29 (Ref)* | 1.00 |  |  |  |  |
| 30-34 years | 0.03 | 0.01 | 0.04 | < 0.001 | 1.6 |
| 35-39 years | 0.04 | 0.02 | 0.05 | < 0.001 | 1.73 |
| 40-44 years | 0.04 | 0.02 | 0.05 | < 0.001 | 1.75 |
| 45-49 years | 0.05 | 0.03 | 0.07 | < 0.001 | 1.63 |
| **Parity** |  |  |  |  |  |
| *No child (Ref)* | 1.00 |  |  |  |  |
| 1 child | -0.02 | -0.04 | 0.00 | 0.042 | 2.14 |
| 2-3 children | -0.05 | -0.07 | -0.04 | < 0.001 | 3.3 |
| 4-5 children | -0.08 | -0.10 | -0.06 | < 0.001 | 3.38 |
| 6 or more children | -0.09 | -0.11 | -0.07 | < 0.001 | 2.94 |

CI: Confidence Interval: Ref: Reference Category

Table S3. Multicollinearity test of parity, Socio-economic status and cervical cancer screening

| **Variable** | **Coefficient** | **95% CI** | | **P-value** | **VIF** |
| --- | --- | --- | --- | --- | --- |
| **Parity** |  |  |  |  |  |
| *No child (Ref)* | 1.00 |  |  |  |  |
| 1 child | -0.01 | -0.03 | 0.01 | 0.303 | 2.14 |
| 2-3 children | -0.03 | -0.04 | -0.01 | 0.006 | 3.25 |
| 4-5 children | -0.03 | -0.05 | -0.01 | 0.001 | 3.15 |
| 6 or more children | -0.02 | -0.04 | 0.00 | 0.033 | 2.68 |
| **Socio-economic status** |  |  |  |  |  |
| *Poor (Ref)* |  |  |  |  |  |
| Middle | 0.02 | 0.01 | 0.03 | 0.003 | 1.18 |
| Rich | 0.07 | 0.06 | 0.08 | < 0.001 | 1.33 |

CI: Confidence Interval: Ref: Reference Category
